# Lactulose Suppresses Osteoclastogenesis and Ameliorates Estrogen Deficiency-Induced Bone Loss in Mice

**DOI:** 10.14336/AD.2019.0613

**Published:** 2019-06-13

**Authors:** Xiao Chen, Zheng Zhang, Yan Hu, Jin Cui, Xin Zhi, Xiaoqun Li, Hao Jiang, Yao Wang, Zhengrong Gu, Zili Qiu, Xin Dong, Yuhong Li, Jiacan Su

**Affiliations:** ^1^Department of Orthopedics Trauma, Shanghai Changhai Hospital, Second Military Medical University, Shanghai, China.; ^2^College of Basic Medicine, Second Military Medical University, Shanghai, China.; ^3^Department of Orthopedics, Jing’ An District Centre Hospital of Shanghai Huashan Hospital, Fudan University, Shanghai, China.; ^4^Jinling high school, Nanjing, Jiangsu, China.; ^5^School of Pharmacology, Second Military Medical University, Shanghai, China.

**Keywords:** lactulose, short-chain fatty acids, gut microbiota, postmenopausal osteoporosis, osteoclastogenesis

## Abstract

Postmenopausal osteoporosis is characterized by excess osteoclastogenesis which leads to net bone loss and brittle fractures. Studies have demonstrated that estrogen deficiency-associated bone loss is microbiota-dependent and could be prevented by probiotics and prebiotics. In this study, we report that orally administered lactulose (20 g/kg, 6 weeks) orally administered significantly inhibited osteoclastogenesis, bone resorption, and prevented ovariectomy (OVX)-induced bone loss in mice. Lactulose increased intestinal *Claudin 2, 3 and 15,* compared to the OVX group, and lowered pro-osteoclastogenic cytokines levels including tumor necrosis factor-α, interleukin(IL)-6, receptor activator of nuclear factor kappa-Β ligand (RANKL), and IL-17 as well as increased the anti-inflammatory cytokine IL-10 in the intestine, peripheral blood, and bone marrow. Lactulose significantly preserved the number of Foxp3^+^ Treg cells in the intestines compared with that in OVX mice. Lactulose altered the composition of intestinal microbiota measured by 16s rDNA sequencing and increased intestinal and serum short-chain fatty acids (SCFAs) levels including acetate, propionate and butyrate which were decreased in OVX mice as measured by gas chromatography. Oral administration of lactulose for 2 weeks significantly lowered the level of bone resorption marker C-telopeptide of type 1 collagen-1 in healthy male young volunteers (aging 20-25 years). In conclusion, lactulose inhibited osteoclastogenesis and bone resorption by altering the intestinal microbiota and increasing SCFAs. Lactulose could serve as an ideal therapeutic agent for postmenopausal osteoporosis.

Bone is continuously remodeling through bone formation by osteoblasts and resorption by osteoclasts [1]. Postmenopausal osteoporosis (PMOP) is a common bone disease leading to brittle fractures and disability. After estrogen levels drop, osteoclastogenesis is overactivated and the bone turnover rate increases, which leads to net bone loss [2, 3]. The primary driver of enhanced osteoclastogenesis is the increased production of receptor activator of nuclear factor-κB ligand (RANKL) and pro-inflammatory cytokines including tumor necrosis factor (TNF) [4, 5].

Recent studies have revealed that the gut microbiota participate in bone remodeling. With increased intestinal permeability, increased exposure to intestinal micro-organisms provides the antigens for T cell activation, and the ensuing systemic immune responses are required for estrogen deprivation-induced bone loss [7, 8]. *Lactobacillus rhamnosus* GG (LGG) or the commercially available probiotic supplement VSL#3 twice weekly reduces gut permeability and intestinal and bone marrow inflammation, and completely protects against bone loss. Moreover, short-chain fatty acids (SCFAs), which are primary gut flora metabolites, have been reported to directly inhibit osteoclastogenesis and prevent postmenopausal and inflammation-induced bone loss [9, 10]. Gut microbiota are involved in the pathogenesis of several multiple metabolic and inflammatory bone diseases, including rheumatoid arthritis and osteoporosis [11]. Probiotics and prebiotics have emerged as therapeutic options for PMOP prevention and treatment.

Lactulose is a synthetic derivative of lactose with one galactose and one fructose. It cannot be digested by enzymes in the mammalian digestive tract but can be used by colonic microflora [12]. It is widely used as an osmotic laxative to treat constipation [13] and benefits host health as a prebiotic [14]. Drinking water containing 10% GOS-Lu (galacto-oligosaccharides derived from lactulose) prevents the development of colorectal cancer by significantly reducing intestinal populations of pro-inflammatory bacterial families, and significantly increasing beneficial populations [15]. A 3-week intervention with lactulose increases the abundance of the hydrogen-producing bacteria *Prevotellaceae* and *Rikenellaceae*, probiotics *Bifidobacteriaceae* and *Lactobacillaceae*, and mucin-degrading bacteria, such as *Akkermansia* and *Helicobacter. *Lactulose decreases the abundance of harmful bacteria, such as *Desulfovibrionaceae* and increases branched-chain SCFAs (BCFAs) [3]. We have previously reported its protective effects on colon inflammation and cerebral ischemia/reperfusion injury by suppressing oxidative stress and inflammation and mobilizing gut microbiota [16-18].

We performed this study to evaluate the effects of lactulose on estrogen withdrawal- induced bone loss. The results show that lactulose protected against ovariectomy (OVX)-induced bone loss in mice and significantly suppressed osteoclastogenesis. Lactulose maintained the intestinal epithelial barrier permeability that decreased after OVX, preserved the number of intestinal Treg cells that decreased significantly after OVX and lowered the pro-inflammatory and osteoclastogenic cytokine levels.

## MATERIALS AND METHODS

### Animals and PMOP model establishment

Female C57BL/6 mice (weight 20-25g; age 8 weeks old) were purchased from Shanghai Slack Co. (Shanghai, China). The mice were maintained under standard Specific Pathogen Free (SPF) conditions at the Animal Experimental Center of Changhai Hospital (SYXK 2015-0017) and fed with sterilized food and autoclaved water. The mice feed was purchased from Shanghai Slack and was comprised of corn (40%), bran (25%), bean cake (30%) and others (5%), including salt, bone powder, and necessary vitamins. The ovariectomy (OVX) procedure was performed as described previously [19]. In brief, a longitudinal dorsal incision was made with the mouse in the ventral recumbency and the abdominal wall muscle fibers were separated with the tips of sharp scissors to locate the ovaries, which were identified and removed. A cotton swab was used to stop the bleeding. The muscle layer and skin were closed with absorbable sutures.

### Animal experimental design

All procedures abided by the guidelines of the Ethics Committee on Animal Experiments of the Second Military Medical University. The 8-week-old mice were randomly divided into different groups of six each. Random numbers were generated using Excel software, and the surgery was performed immediately. Sham group: sham-operated mice treated with vehicle; Sham + Lac group: sham-operated mice treated with lactulose (Abbott Biologicals B.V., Best, the Netherlands); the OVX group: ovariectomized mice treated with vehicle; and the lactulose (Lac) group: ovariectomized mice treated with lactulose. Mice in the sham or OVX groups were gavaged with normal saline every day. The lactulose-treated mice were gavaged with 20 g/kg lactulose per day after surgery. After 6 weeks, the mice were sacrificed and the femur, arterial blood, distal 3cm of the colon and feces were collected. Blood was centrifuged at 3,000 rpm for 5 min, and the supernatant was stored at -80?. Feces were stored immediately in liquid nitrogen.

### Effects of lactulose on bone remodeling in humans

Healthy individuals (male, n=7) received an oral administration of 7.5 g/day lactulose (Abbott Biologicals) once per day for 14 days. Inclusion criteria: healthy young male 20-25 years of age; Exclusion criteria: (1) diarrhea in the past 3 months; (2) inflammatory bowel disease; (3) chronic inflammatory bone diseases, including rheumatoid arthritis or seronegative spondylo-arthropathies; (4) lactose intolerance; (5) galactosemia; (6) allergic to lactulose; and (7) lactulose used in the past 3 months. All procedures were approved by the Ethical Committee of Changhai Hospital. Informed consent was obtained from all subjects. Serum was sampled on day 1 and 14 and analyzed for C-terminal collagen type I cleavage products (CTX-1) as a marker for bone resorption and osteocalcin (OCN) as a marker for bone formation by enzyme-linked immunosorbent assay (ELISA).

### Histomorphometric analysis

Bone histomorphometric analyses of the distal femoral metaphysis were performed as described previously [20]. Briefly, the femurs were fixed and decalcified for two weeks. Then sections (4 μm thick) were prepared with a microtome and stained with hematoxylin and eosin. Tartrate-resistant acid phosphatase (TRAP) staining was used to evaluate osteoclastogenesis.

### Immunohistological analysis

The bone and intestinal sections were immersed in 10 mM citrate buffer for antigen retrieval. The bone sections were blocked and incubated with the mouse anti-OCN (1:1,000, Abcam, Cambridge, MA, USA). The intestinal sections were blocked and incubated with anti-Claudin-2 overnight at 4°C. After washing with PBS, the sections were incubated with goat anti-mouse antibody (1:1000, Abcam) or rabbit anti-mouse antibody (1:1000, Abcam) at room temperature for 1 hour. The osteoid matrix areas were measured using Image J software (National Institutes of Health, Bethesda, MD, USA). Five microscopic fields were chosen randomly from each sample.

### Micro-computed tomography analysis

Micro-computed tomography (CT; Skyscan 1172, Bruker, Antwerp, Belgium) was used to analyze the trabecula of the femur (80 kV, 124 μA, resolution 8 μm). The structural parameters of the metaphyseal region of the proximal femur were analyzed with the *built-in* software, including bone mineral density (BMD), bone surface/bone volume (BS/BV), bone surface area/total volume (BS/TV), bone volume/total volume (BV/TV), trabecular number (Tb.N) and trabecular space (Tb.Sp).

### Immunofluorescence staining

The distal 3 cm of the intestine was collected and fixed in 4% paraformaldehyde. The sections were deparaffinized, washed, blocked, and incubated with primary antibody (goat anti-mice FOXP3 at 1:100, Abcam) and then with secondary antibodies. Nuclei were stained with DAPI. Three microscopic fields were randomly chosen from each slide and analyzed with the Image Pro Plus to calculate the number of positive cells.

### Enzyme-linked immunosorbent assay 

Bone marrow, small intestine and serum were collected and analyses for IL-6, TNF-α, RANKL, IL-17, and IL-10 by ELISA kits (R&D Systems, Minneapolis, MN, USA) according to the manufacturer’s directions. Rodent serum tartrate-resistant acid phosphatase 5b (TRAcp-5b) and OCN were measured by rodent-specific ELISA (Immunodiagnostic Systems, Scottsdale, AZ, USA).

### Real-time quantitative polymerase chain reaction (RT-qPCR)

RNA levels were quantified by RT-qPCR. The mRNA levels of *Claudin 2*, *Claudin 3* and *Claudin* 15 were measured in the small intestine and colon. All the primers used were designed by Primer Express Software v3.0.1 (Applied Biosystems, Foster City, CA, USA). Changes in relative gene expression were calculated using the 2-^Δ^CT method with normalization to 18S rRNA. The primers for *Cldn2* were: 5′-TCTCAGCCCTGTTTTCTTTGG-3′ (forward) and 5′-GGCGAGCAGGAAAAGCAA-3′ (reverse); for *Cldn3*, 5′-TCATCACGGCGCAGATCA-3′ (forward) and 5′-CTCTGCACCACGCAGTTCA-3′ (reverse); and for *Cldn15*, 5′-GGCGGCATCTGTGTCT TCTC-3′ (forward); and 5′-TGGTGGCTGGTTCCTCC TT-3′ (reverse).

### Fecal 16s rDNA sequencing

Stool samples were freshly collected and stored at -80? before use. DNA was extracted from 0.18 to 0.22 g of stool using a QIA amp DNA Stool Mini Kit (Qiagen, Valencia, CA, USA). The DNA was recovered with 30 mL of AE buffer (10 mM Tris-Cl, 0.5 mM EDTA, pH 9.0; Qiagen). The 16S ribosomal RNA (rRNA) gene was analyzed to evaluate the bacterial diversity, using the Illumina Hiseq (Novogene Bioinformatics Technology Co., Sacramento, CA, USA).

### SCFAs measurement

Fecal and serum SCFAs levels were measured. Briefly, 100 mg of frozen fecal sample or 50 µl serum was added to polypropylene tubes and kept in a cool environment. The samples were acidified and vortexed for 1 min. Diethyl ether was added, vortexed for 1 min, and centrifuged for 3 min at 4°C. The organic phase was transferred to a 2 ml gas chromatography (GC) vial, and a GC mass spectrometric (GCMS) analysis was performed.

### Statistical analysis

**Figure 1.**
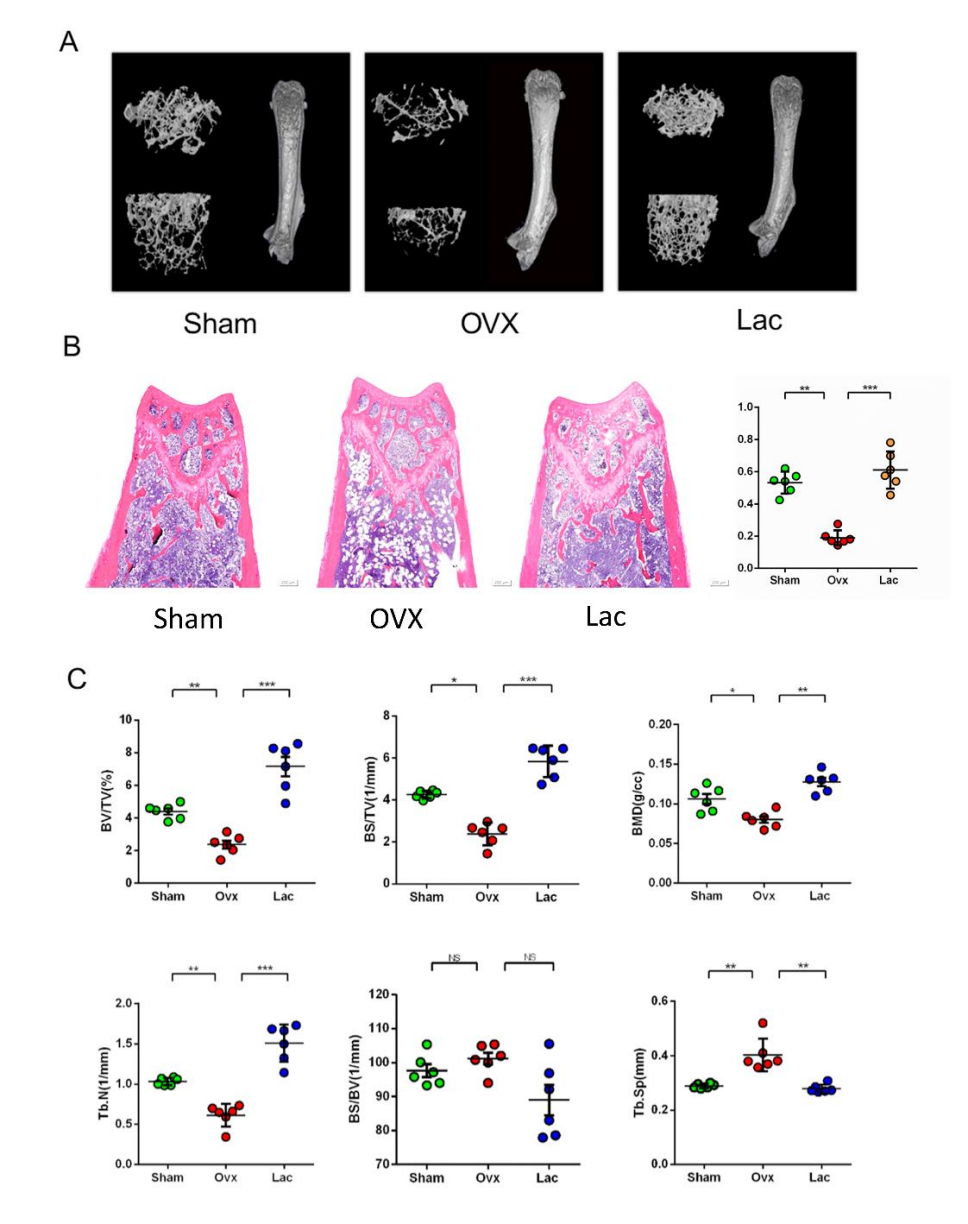
Lactulose prevented bone loss induced by ovariectomy in mice. (A) Representative μCT analysis of the distal femur. (B) Representative H&E staining of distal femoral sections and quantification of the trabecular area from each group 6 weeks after the operation of H&E staining. Scale bar: 200 μm. (C) Calculations of bone value / total value (BV/TV), bone surface area /total value (BS/TV), bone mineral density (BMD), trabecular number (Tb.N), bone surface area / bone value (BS/BV), trabecular space (Tb.Sp). Data are expressed as mean ± SEM. *P < 0.05, **P < 0.01, and ***P < 0.001 compared with the corresponding group.

**Figure 2.**
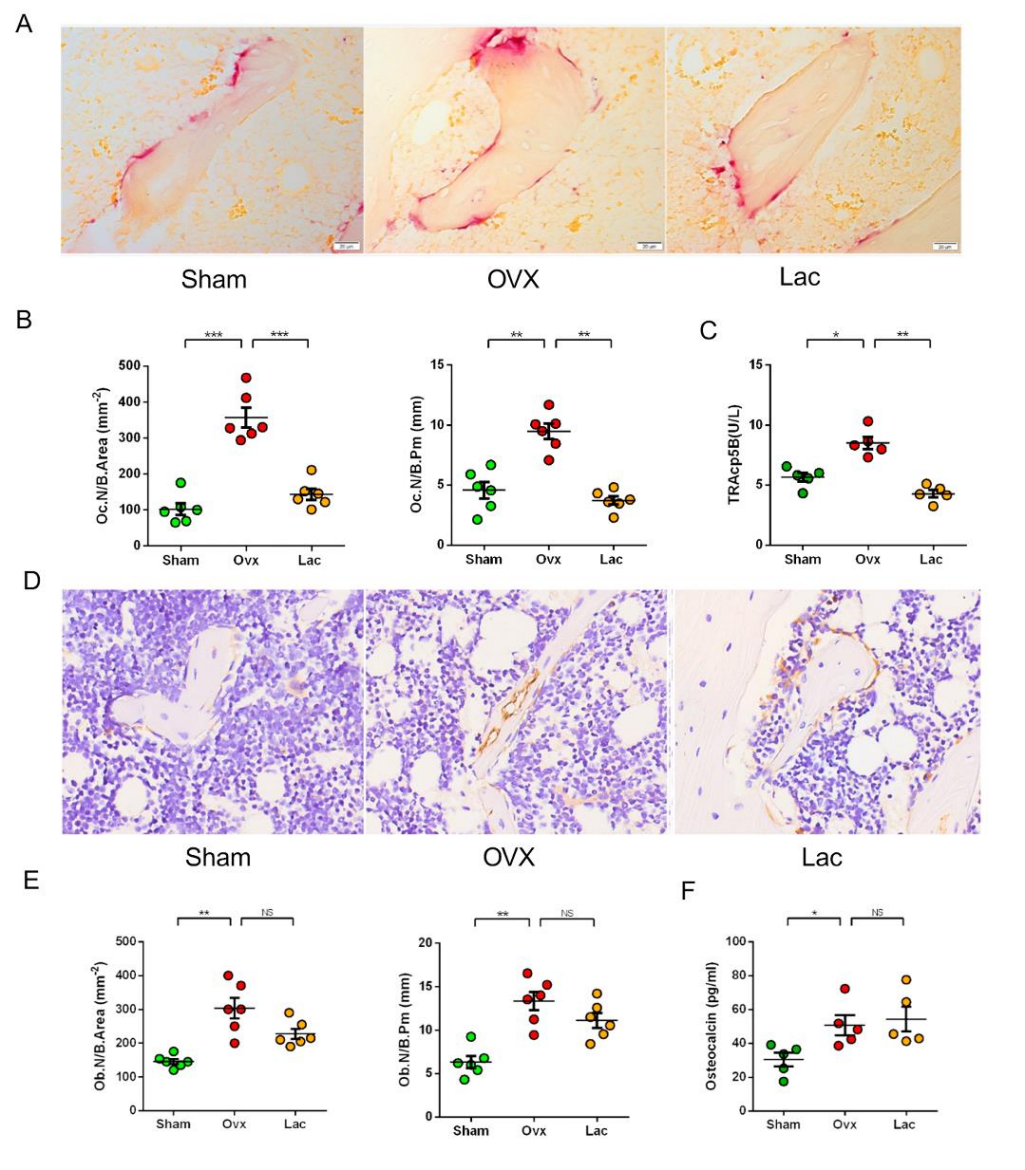
Lactulose suppressed osteoclastogenesis. (A) Tartrate-resistant acid phosphatase-stained sections of the distal femur. Scale bar: 20 μm. (B) Oc.N / B.Area. and Oc.N / B.pm. (C) Serum levels of TRAcp-5b. (D) Osteocalcin stained sections of the distal femur. Original magnification ×40. Scale bar: 20 μm. (E) Ob.N / B.Area. and Ob.N / B.pm. (F) Serum levels of osteocalcin. n= 5 mice per group in all panels. Data are expressed as mean ± SEM. *P < 0.05, **P < 0.01, and ***P < 0.001 compared with the corresponding group.

**Figure 3.**
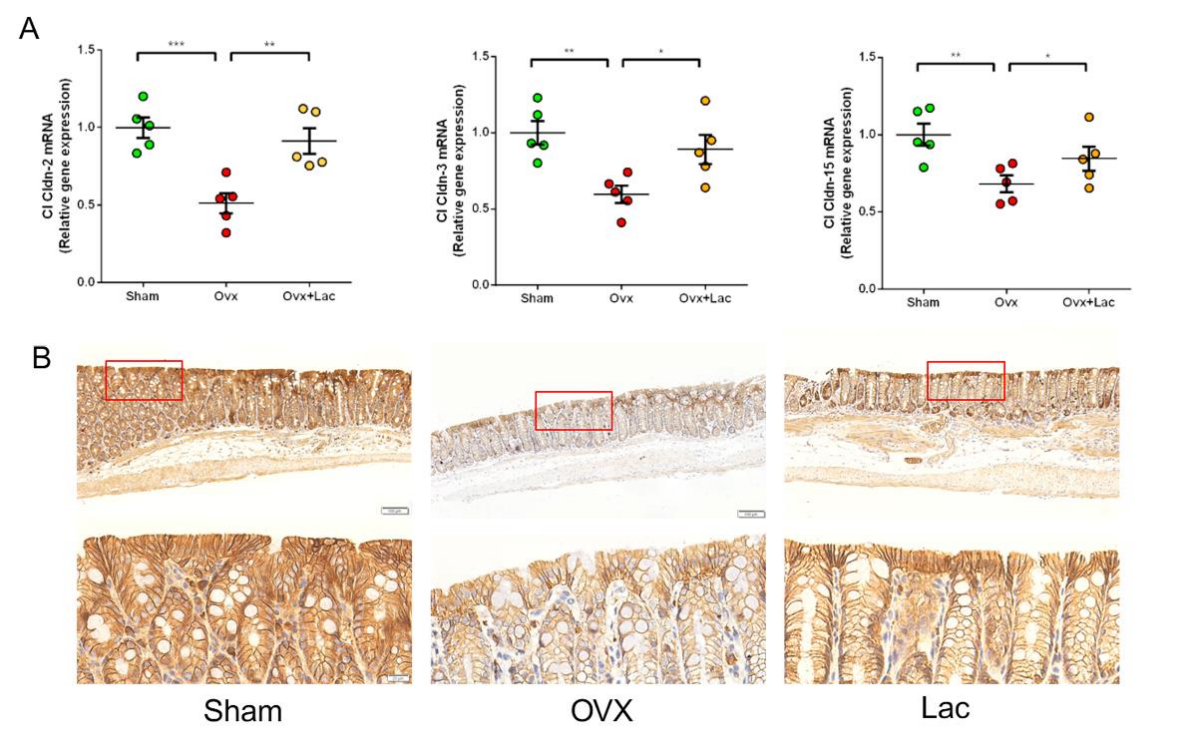
Lactulose reduced intestine permeability after OVX. (A) Transcript levels of the tight junction proteins *claudin 2*, *claudin 3* and *claudin 15* of Sham, OVX and OVX mice treated with lactulose in the small intestine. (B) Immunohistological analysis of claudin 2 expressions in small intestine of Sham, OVX and OVX mice treated with lactulose. (Scale bar: 200 or 20 μm). Data are expressed as mean ± SEM. *P < 0.05, **P < 0.01, and ***P < 0.001 compared with the corresponding group.

**Figure 4.**
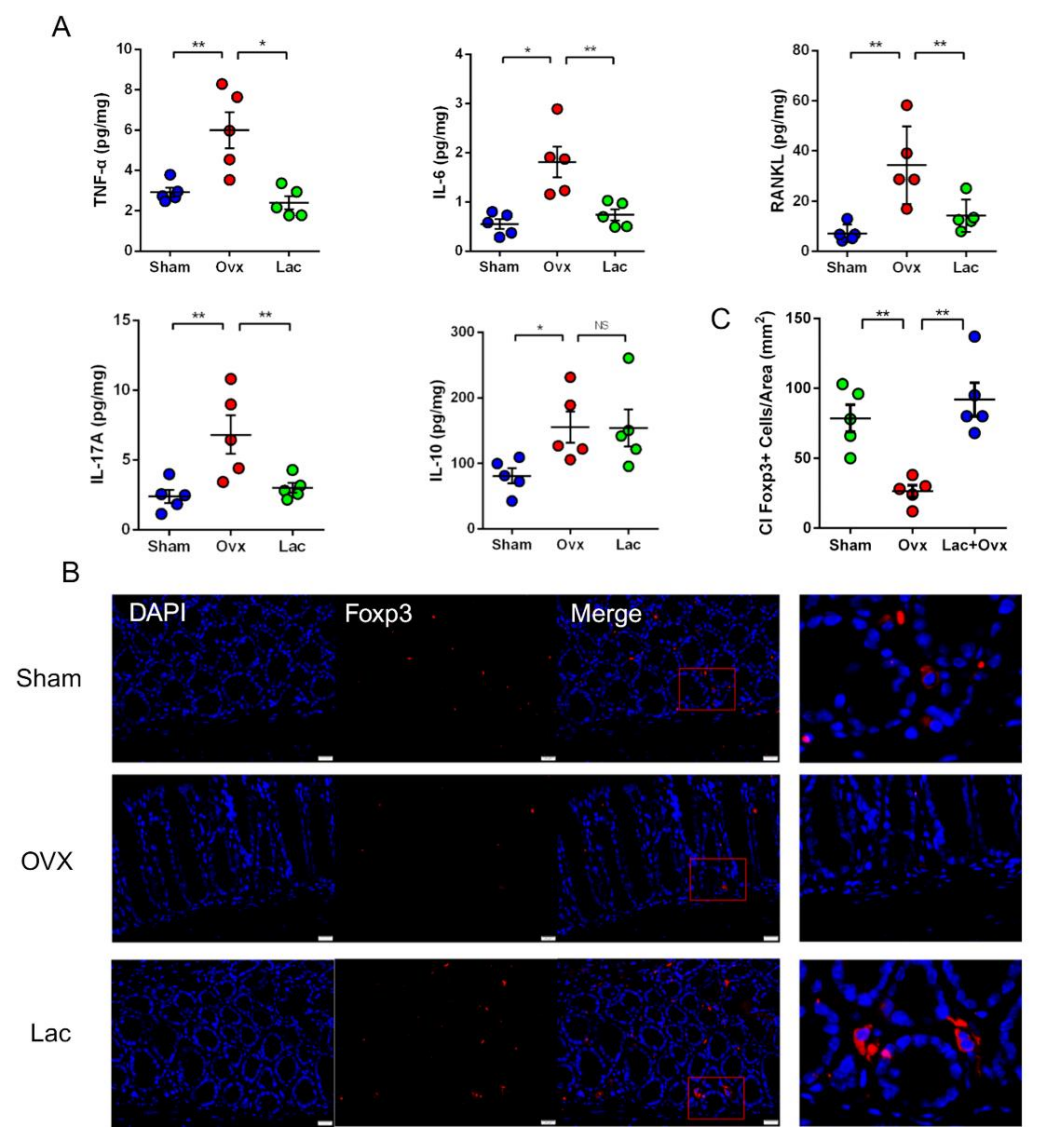
Lactulose inhibited OVX-induced pro-inflammatory cytokines. (A) Levels of the inflammatory cytokines TNF-α, IL-6, RANKL, and IL-17 and anti-inflammatory cytokine IL-10 in the small intestine of Sham, OVX and OVX mice treated with lactulose. (B) Immunofluorescent analysis of Foxp3^+^ cells in the intestine from Sham, OVX and OVX mice treated with lactulose small. Scale bar: 20 μm. (C) Calculation of Foxp3^+^ cells/Area. n = 5 mice per group in all panels. Data are expressed as mean ± SEM. *P < 0.05, **P < 0.01, and ***P < 0.001 compared with the corresponding group.

**Figure 5.**
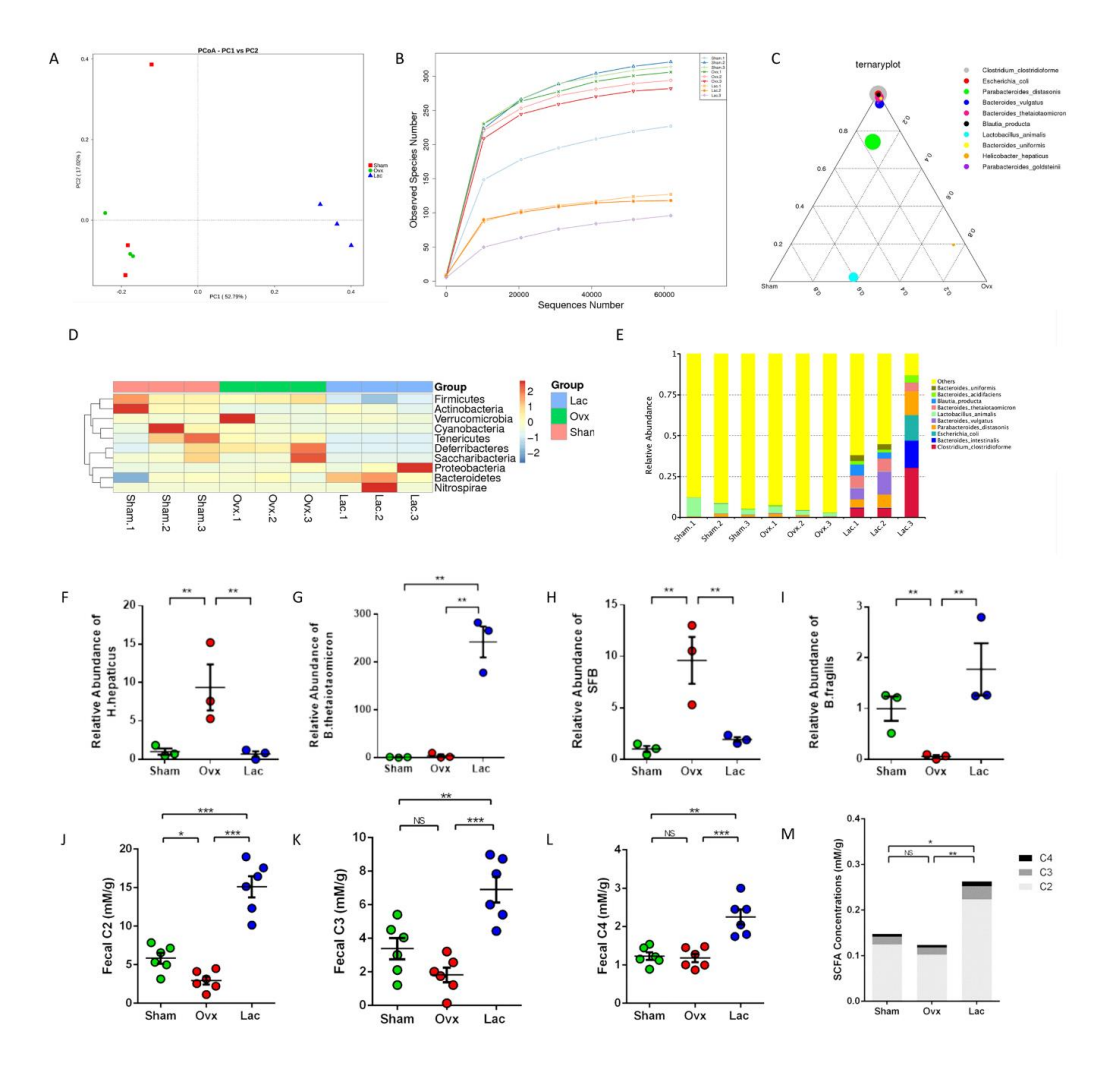
Lactulose altered gut microbiota and increased SCFA production. (A) PCoA analysis. (B) Observed Species Number in different sequence number. (C) Ternary plot of different groups. (D) Taxonomy distribution at the genus level in different samples. (E) Top 10 enriched species in all groups. (F-I) Significantly differed species in the different groups; (J-L) The fecal SCFA levels of each group. (M) The serum SCFA levels of each group. Data are expressed as mean ± SEM. *P < 0.05, **P < 0.01, and ***P < 0.001 compared with the corresponding group.

All data are presented as the mean ± standard error. Levene’s test was performed to determine the homogeneity of variance in the different groups. If Levene’s test was not significant, then comparisons between different groups were calculated using a *t*-test or one-way analysis of variance followed by Tukey’s post hoc test (data in Fig. 1-3). If Levene’s test was significant, then differences between multiple groups were compared with the Kruskal-Wallis test followed by the Dunn-Bonferroni post-hoc method for multiple comparisons (data in Fig. 4 and 5). A p-value < 0.05 was considered significant.

## RESULTS

### Lactulose prevents bone loss induced by ovariectomy in mice

To investigate the effects of lactulose on OVX-induced bone loss, we administered lactulose orally to OVX mice. The higher concentration (40 g/kg) caused diarrhea and the optimal dose (20g/kg) was determined. The effects of lactulose on the structure of distal femur trabecular bone were analyzed by micro-CT (Fig. 1A) and hematoxylin and eosin staining showed a reduced trabecular bone area in the OVX group and retention in the distal femur of the lactulose group (Fig. 1B). Lactulose significantly increased the distal femoral BV/TV, BS/TV, Tb.N and bone mineral density (BMD) relative to the OVX group (Fig. 1C). The femoral cortex was also measured, but no significant differences were detected between the groups (Supplementary Fig.1).

We also determined whether lactulose could affect bone mass in the sham mice. After lactulose treatment, trabecular area, as shown by H&E staining and BV/TV, BS/TV, Tb.N, and BMD increased significantly (Supplementary Fig. 2). These results indicate that lactulose affects bone metabolism independently of estrogen.

### Lactulose suppressed osteoclastogenesis in vivo

We examined the effects of lactulose on osteoclasto-genesis *in vivo*. TRAP staining revealed a significant increase in the number of osteoclasts after OVX, whereas lactulose-treated mice had an equivalent number of osteoclasts compared to those of the sham controls (Fig. 2A-B). Serum TRAcp5b increased significantly in OVX mice while lactulose lowered the level (Fig. 2C). The immunohistological analysis of OCN revealed an increase in the number of osteoblasts in OVX mice relative to the sham group (Fig. 2D-E). Although lactulose tented to reduce the number of osteoblasts, the difference was not significant compared with OVX mice. Serum OCN level increased in OVX mice and lactulose-treated mice, compared with the sham controls (Fig. 2F). No significant difference was found between the lactulose and OVX groups. Calcein staining showed that the mineral apposition rate (MAR) and bone formation rate (BFR) increased after OVX, and lactulose administration significantly reduced MAR and BFR in OVX mice (supplementaty Fig. 3A-B). Lactulose reduced serum TRAcp5b level in the sham mice (supplementaty Fig. 4). A 14-day supplementation of lactulose (7.5g/day) in healthy male volunteers (Age 21 ± 3 years) significantly decreased serum CTX-1, while the OCN level was not significantly affected (supplementaty Fig. 3C). Taken together, the results suggest that lactulose treatment suppresses osteoclastogenesis and lowers bone turnover rate *in vivo*.

### Lactulose maintains intestinal permeability after OVX 

To explore the effects of lactulose on the intestinal barrier, we measured transcript levels of *Claudin* family members in the intestine, which are the principal members of gap junction proteins that maintain intestinal barrier integrity. OVX mice had lower *Claudin 2*, *3*, and *15* mRNA levels in the intestine compared to the sham controls (Fig. 3A). Lactulose prevented the decrease in *Claudin 2*, *3*, and *15 *mRNA levels. The immunohistochemistry results demonstrated that Claudin 2 expression decreased after OVX and lactulose increased the expression of Claudin 2 relative to that in the OVX group (Fig. 3B). These results indicate that estrogen deprivation jeopardizes the integrity of the intestinal epithelium and increases permeability, while lactulose restores the epithelial barrier function and decreases permeability.

### Lactulose inhibits OVX-induced inflammation

To further explore the effects of lactulose on inflammation, we investigated the changes in intestinal pro-osteoclastogenic cytokines. The levels of TNF-α, IL-6, RANKL, and IL-17 increased after OVX, while IL-10 decreased. Administering lactulose lowered TNF-α, IL-6, RANKL, and IL-17 levels and increased IL-10 levels (Fig. 4A). The levels of TNF-α, IL-6, RANKL, and IL-17 in the intestine, bone marrow (BM) (supplementary Fig. 5A) and peripheral blood (supplementary Fig. 5B) changed synergistically. Foxp3 is a cellular marker of activated Treg cells. Immunofluorescence revealed a significant decrease in the number of Foxp3^+^ Treg cells in the intestine after OVX. Lactulose preserved the number of Foxp3^+^ Treg cells number in OVX mice relative to OVX mice (Fig. 4B).

### Lactulose altered gut microbiota and increased SCFAs production

The principal component analysis reflected that the structure of the gut microbiota did not change significantly after OVX. However, lactulose significantly changed the composition of the gut microflora (Fig. 5A). Similar α-diversity indices, including observed species number, ACE, and Shannon were observed in the sham and OVX groups (Fig. 5B, S6A-B). However, α-diversity decreased in mice treated with lactulose (Fig. 5B). The ratio of *Firmicutes* and *Bacteroidetes* decreased significantly after lactulose administration (Fig. 5D). The three groups had different dominant genera (Supplementary Fig. 6C), indicating that the composition of gut microbiota was also changed in OVX mice. *Clostridium clostridioforme, Escherichia coli, Para-bacteroides distasonis, Bacteroides vulgatus, Bacteroides thetaiotaomicron, Blautia producta, Bacteroides uniformis, *and *Barabacteroides goldsteinii* became dominant species with over 50% abundance in the total gut microbiota of the lactulose treated OVX mice (Fig. 5C, E). The quantity of *Helicobacter hepaticus *in the OVX group was 9.75-fold that in the sham group (Fig. 5F). In addition, two important organisms, such as segmented filamentous bacteria (SFB) and *Bacteroides fragilis*, related to the proliferation and differentiation of Th17s and Tregs increased and decreased, respectively, in OVX mice, and decreased and increased in response to lactulose, respectively (Fig. 5H-I).

**Figure 6.**
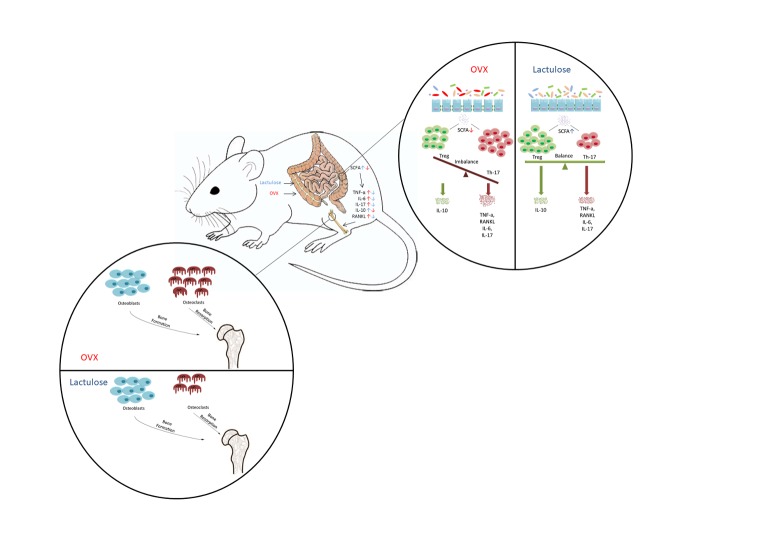
Graphical abstract. Estrogen withdrawal results in reduced fecal SCFA, increased systemic pro-inflammatory cytokines levels, excessive osteoclastogenesis, and significant bone loss. Lactulose increases SCFA production, lowers osteoclastogenic cytokines levels, inhibits osteoclastogenesis and ameliorates bone loss.

We examined serum and fecal SCFAs levels after lactulose administration by gas chromatography. C2 (acetate) levels decreased significantly after OVX, and lactulose intake significantly increased C2, C3 (propionate), and C4 (butyrate) concentrations in serum and feces of mice (Fig. 5J-M) (Fig. 6). Taken together, these results show that OVX slightly altered the gut microbiota and that the lactulose treatment significantly changed intestinal flora composition and promoted SCFAs production.

## DISCUSSION

Our data suggest that lactulose suppresses osteoclastogenesis and ameliorates bone loss after estrogen deprivation in mice. A 2-week lactulose administration in healthy young people significantly lowered the bone resorption marker CTX-1. The mechanism was to inhibit excess osteoclastogenesis involving increase in fecal SCFAs levels, altered gut microbiota, maintenance of intestinal permeability and suppression of pro-inflammatory cytokines. Thus, lactulose could serve as a promising candidate for PMOP prevention and treatment.

Lactulose is a synthetic disaccharide composed of fructose and galactose that cannot not be digested or absorbed by humans. Lactulose is widely used in the treatment of constipation and hepatic encephalopathy [21, 22]. It is metabolized in the colon by bacterial flora to SCFAs, including lactic acid and acetic acid. These compounds partially dissociate, acidifying the colonic contents [23, 24]. The effects of lactulose on mineral metabolism have been observed for a long time. Schaafsma et al. [25] performed a small sample clinical trial and reported that lactulose stimulates calcium absorption in postmenopausal women. Similar results have been reported in animal models [26, 27]. These studies suggest a potential bone protective effect for lactulose. In addition, we previously reported that lactulose has significant antioxidant and anti-inflammatory effects against inflammatory bowel disease and cerebral ischemia-reperfusion injury [16, 17], suggesting an immune regulatory function and a wider potential clinical use. In this study, we revealed that lactulose administration significantly increases bone volume and improves the inflammatory condition both in the BM and intestines of OVX mice.

PMOP is closely related to chronic systemic inflammation with increases in pro-inflammatory cytokines, such as TNF-α and IL-17 [28, 29]. The change in immune status, particularly the excessive increases in inflammatory cytokines detected in PMOP patients, plays an important role in excess osteoclastogenesis [30-32]. Th-17 cells and elevated IL-17 levels can aggravate bone loss after menopause [33, 34]. IL-17R knockout or anti-IL-17 antibody administration can reverse the bone mass loss in OVX mice [35, 36]. Along with RANK and RANKL, the roles of cytokines, including TNF-α, IL-1, and IL-6 as stimulators of osteoclastogenesis are well established [3, 37, 38]. TNF-R1 initiates the NF-κB and MAPK pathways and IL-6R activates JAK-STAT signaling in osteoclast precursors, which promotes osteoclastogenesis [3, 39]. IL-17 receptor signaling in osteoblasts/osteocytes mediates parathyroid hormone-induced bone loss and enhanced osteocytic RANKL production [40]. In contrast, Treg cells inhibit the differentiation of osteoclasts from peripheral blood mononuclear cells [41]. However, the exact originating locations of these pro-inflammatory and pro-osteoclastic cytokines are still unknown.

The intestinal barrier is vital for intestinal and systemic immune responses. A tight barrier is formed by tight junction proteins, involving the Claudin protein family, between epithelial cells to selectively limit diffusion of luminal toxins and antigens through the mucosa [42]. Nevertheless, a breach in cell-to-cell adhesion, also called a “leaky gut”, could result in bacterial translocation and toxin invasion, leading to an intestinal inflammatory response [43]. An intestinal barrier disturbance is related to various inflammatory diseases, including Crohn’s disease and ulcerative colitis [44]. Our results show that intestinal permeability increases after estrogen withdrawal, which allows many antigens entering the epithelial submucosa to modulate T cell behavior both locally and systemically [8], which is probably an important reason for the increase in pro-inflammatory cytokines after menopause. In this study, we found that lactulose treatment restored the decrease in Claudin proteins after OVX, thus guarding the intestinal barrier. OVX mice had higher levels of the pro-inflammatory cytokines TNFα, IL-6, RANKL, and IL-17 in the intestine as well as a lower level of the anti-inflammatory cytokine IL-10. The immunofluorescence results showed that the number of Treg cells decreased significantly in OVX mice and was maintained by the lactulose treatment. We propose that the intestine serves as an important source of pro-inflammatory and osteoclastogenic cytokines after estrogen withdrawal.

The gut microbiota have been reported to play an important role in POMP [45]. In mice, estrogen deficiency increases gut permeability, expands Th17 cells, and upregulates the osteoclastogenic cytokines TNF-α, RANKL, and IL-17 in the small intestine and the BM [46]. Probiotics such as LGG reduce gut permeability, dampen intestinal and BM inflammation, and completely protect against bone loss. We investigatedbacterial taxonomy via 16S rDNA sequencing. In OVX mice, the richness, α-diversity, and quantity of typical probiotics including *Lactobacillus* and *Bifidobacterium* did not change significantly, which accords with previous studies [47, 48]. Two important species, such as SFB and *B. fragilis*, which are related to proliferation and differentiation of Th17 cells and Treg cells increased and decreased respectively in OVX mice. The relationship between estrogen and gut microbiota has been investigated previously [49]. Under healthy conditions, homeostasis is maintained through interplay between the intestinal epithelial barrier, the intestinal microbiota, and the host immune system, with inhibition of intestinal pathogens. In postmenopausal women, estrogen deficiency alters intestinal microbial composition and structure, leading to decreased microbial diversity [49-51]. Intestinal pathogens intrude into a host with compromised intestinal barriers and ignite an immune response, ultimately promoting osteoclastic bone resorption and continual bone loss in PMOP [49]. Wang et al. [19] reported that the proportion of *Firmicutes* in PMOP women was significantly higher and that *Bacteroidetes* was significantly lower than that in normal controls. In summary, the intestinal microbiota, host immune system, and intestinal epithelial barrier are interconnected. Changes in one lead to changes in the others.

After a 6-week lactulose treatment,* Bacteroides thetaiotaomicron*, *Bacteroides uniformis*, *Para-bacteroides distasonis,* and *Clostridium clostridioforme* became dominant species in the mouse gut. Interestingly, these species are not probiotics [14]. The impact of lactulose on changes in microbiota composition in mice and humans varies in different studies. C57BL/6J mice were fed diets supplemented with lactulose (0%, 5%, and 15%) for 2 weeks and the results showed that luminal content was mostly dominated by *Firmicutes, Actinobacteria*, and *Bacteroidetes*, while the mucus was dominated by *Firmicutes, Proteobacteria*, and *Bacteroidetes*. The abundance of *Actinobacteria* increased significantly, and *Proteobacteria* was the most abundant phylum (~50%) in the mucus after a high-lactulose treatment [39]. In humans, changes in the *Lactobacillus* spp. population in response to lactulose intake are conflicting. Terada et al. [52] reported a reduction of *Lactobacillus *spp. in volunteers receiving 3 g lactulose once daily for 14 days. In contrast, Ballongue et al. [53] observed that using 20 g lactulose/day for 4 weeks increased *Lactobacillus*, *Bifidobacteria*, and *Streptococcus* but decreased *Bacteroides*, *Clostridia* and Coliforms. The reasons for the discrepancies are very complex and probably involve dosages, duration, diets and animal strains. In our study, the relative quantity of SFB, *B. fragilis,* and* H. hepaticus* changed significantly in OVX mice compared to sham mice. Among them, *H. hepaticus* showed an increase of 9.75-fold after OVX. *H. hepaticus* could induce inflammatory bowel disease in immune-deficient mice and lead to femoral bone loss [54, 55]. One study showed that *H. hepaticus* colonization in wild-type mice promotes differentiation of RORγt-expressing microorganism-specific iTreg cells in the large intestine and in IL10-deficient mice expanded colitogenic Th17 cells [56]. *H. hepaticus* is likely to participate in T cell regulation in the intestine, but this requires further study.

SCFAs are major intestinal microbiota fermentation products that are chemically composed of a small hydrocarbon chain and a carboxylic acid moiety [57]. Acetic, propionic and butyric acids are the three most studied SCFAs [58]. They function as a link between the gut flora and immune system by regulating various types of intestinal epithelial cells, and leukocyte development, survival, and function through downstream signaling of G protein-coupled receptors (GPR41, GPR43, GPR109a, and Olfr78) [59, 60]. SCFAs regulate the intestinal immune balance and are related to a series of inflammatory and metabolic diseases including in?ammatory bowel disease, obesity and rheumatoid arthritis [10]. SCFAs have been reported to inhibit osteoclastogenesis directly by promoting glycolysis [9]. In our study, we found a significant reduction of fecal SCFAs in OVX mice and lactulose treatment significantly increased serum and fecal SCFAs concentrations.

Our results show that the gut and bone are closely coupled. After estrogen withdrawal, the intestinal barrier was jeopardized and the interaction between the intestinal microbiota and the host intestinal immune system was altered. Anti-inflammatory Treg cells decreased and pro-inflammatory cytokines increased in the intestine. The blood circulation acts as a bridge between the gut microbiota and bone [61]. Cytokines, T cells, and bacterial metabolites, such as SCFAs, can be transported into the BM to regulate bone remodeling. Lactulose inhibits osteoclastogenesis and protects against bone loss after estrogen deficiency by maintaining the homeostasis of the intestine barrier, host intestine immune system, and gut microbiota.

## Supplementary Materials

The Supplemenantry data can be found online at: www.aginganddisease.org/EN/10.14336/AD.2019.0613.
