# Supplementary material for: Lactulose Suppresses Osteoclastogenesis and Ameliorates Estrogen Deficiency-Induced Bone Loss in Mice
Source: Aging Dis. 2019 Jun 13;11(3):629–41. doi: 10.14336/AD.2019.0613 (PMC7220299; doi:10.14336/AD.2019.0613)
Supplement: Supplementary file 1 — The Supplemenantry data can be found online at: www.aginganddisease.org/EN/10.14336/AD.2019.0613. [file AD-11-3-629-s.pdf]

SUPPLEMENTARY DATA

Lactulose Suppresses Osteoclastogenesis and Ameliorates Estrogen Deficiency-Induced Bone Loss in Mice

Xiao Chen[1,#], Zheng Zhang[2,#], Yan Hu[1,#], Jin Cui[1], Xin Zhi[2], Xiaoqun Li[2], Hao Jiang[1], Yao Wang[1], Zhengrong Gu[3], Zili Qiu[4], Xin Dong[5], Yuhong Li[1], Jiacan Su[1,2*]

[1]Department of Orthopedics Trauma, Shanghai Changhai Hospital, Second Military Medical University, Yangpu District, Shanghai, China. [2]College of Basic Medicine, Second Military Medical University, Shanghai, China. [3]Department of Orthopedics, Jing' An District Centre Hospital of Shanghai Huashan Hospital, Fudan University, Shanghai, China. [4]Jinling high school, Nanjing, Jiangsu Province China. [5]School of Pharmacology, Second Military Medical University, Yangpu District, Shanghai, China.

© 2019. Chen X et al. Published online at http://www.aginganddisease.org/EN/10.14336/AD.2019.0613

SUPPLEMENTARY DATA

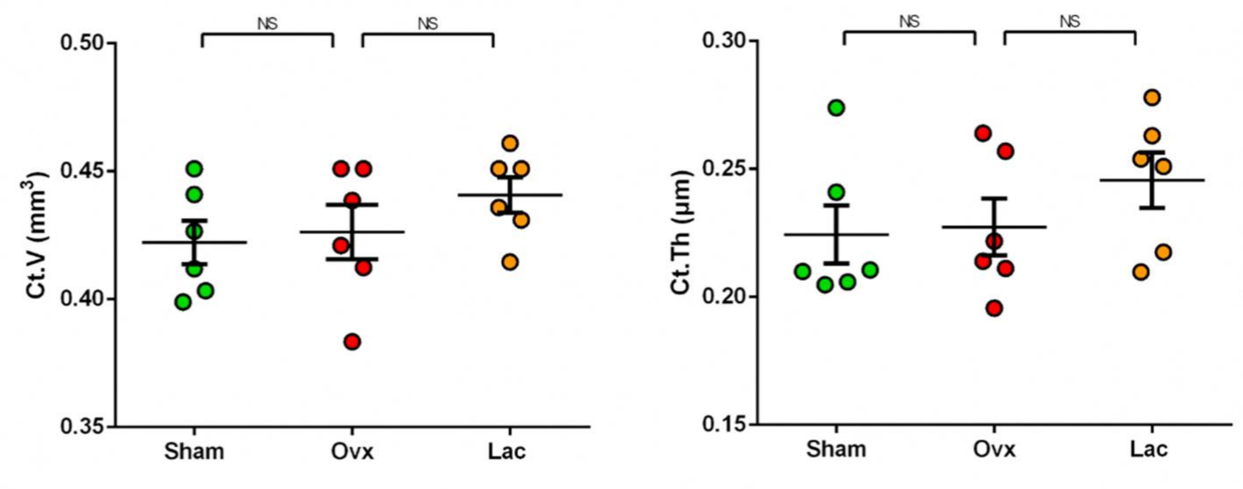

Supplementary Figure 1. **Analysis of femoral cortical bone.** No significant difference was found between groups. Data are expressed as mean ± SEM.

© 2019. Chen X et al. Published online at http://www.aginganddisease.org/EN/10.14336/AD.2019.0613

SUPPLEMENTARY DATA

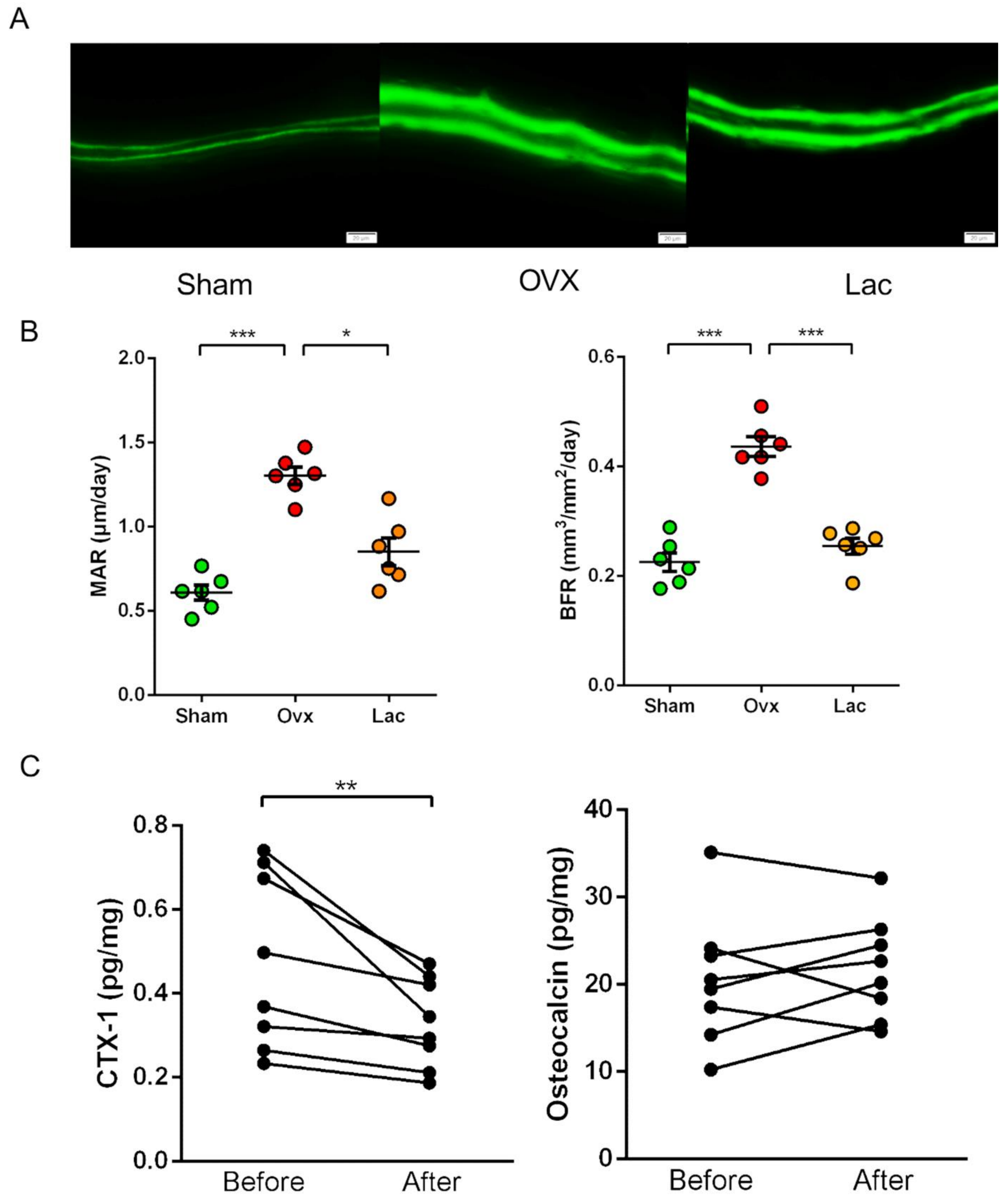

Supplementary Figure 2. Lactulose increased bone mass in Sham mice. (A) Representative μCT analysis of the distal femur. (B) Representative H&E staining of distal femoral sections and quantification of the trabecular area from each group 6 weeks after the lactulose administration. Scale bar: 200 μm. (C) Calculations of bone value / total value (BV/TV), bone surface area /total value (BS/TV), bone mineral density (BMD), trabecular number (Tb.N), bone surface area / bone value (BS/BV), trabecular space (Tb.Sp). Data are expressed as mean ± SEM. **P* < 0.05, ***P* < 0.01, and ****P* < 0.001 compared with the corresponding group.

© 2019. Chen X et al. Published online at http://www.aginganddisease.org/EN/10.14336/AD.2019.0613

SUPPLEMENTARY DATA

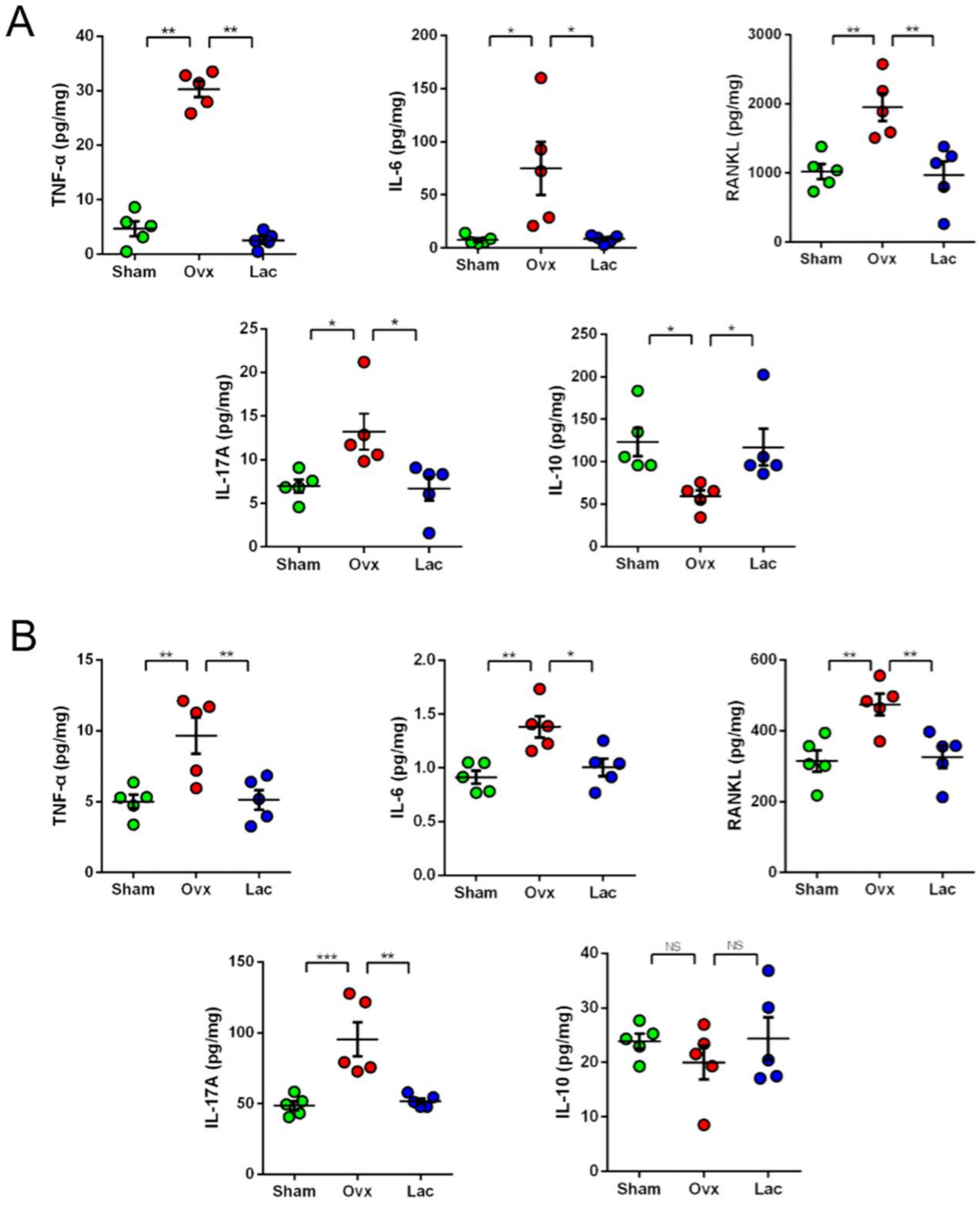

Supplementary Figure 3. Lactulose inhibited osteoclastogenesis and bone remodeling. (A) Calcein staining images. (B) MAR and BFR analysis. (C) Measurement of serum CTX-1 and OCN before and after lactulose administration in healthy volunteers. Serum Crosslaps bone resorption (CTX-1) and formation (OCN) marker measured by ELISA before and after two weeks of lactulose treatment (7.5g/day) in healthy control human subjects. Data are expressed as mean ± SEM. **P* < 0.05, ***P* < 0.01 and ****P* < 0.001 compared with the corresponding group.

© 2019. Chen X et al. Published online at http://www.aginganddisease.org/EN/10.14336/AD.2019.0613

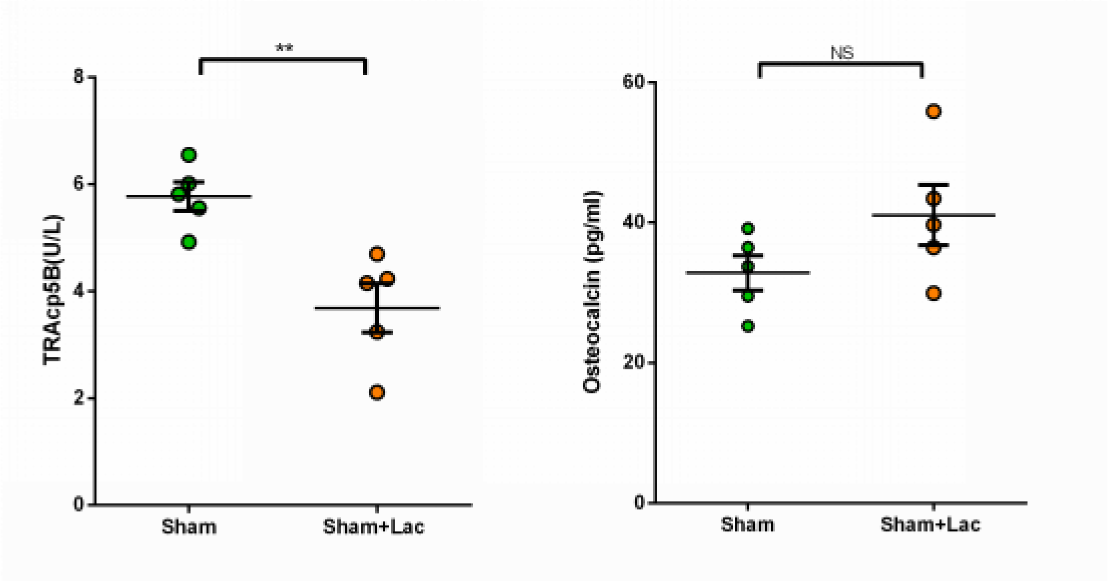

Supplementary Figure 4. Lactulose lowered serum TRAcp5b level in Sham mice. Serum levels of TRAcp-5b and osteocalcin. n= 5 mice per group in all panels. Data are expressed as mean ± SEM. **$P < 0.01$compared with the corresponding group.

© 2019. Chen X et al. Published online at http://www.aginganddisease.org/EN/10.14336/AD.2019.0613

SUPPLEMENTARY DATA

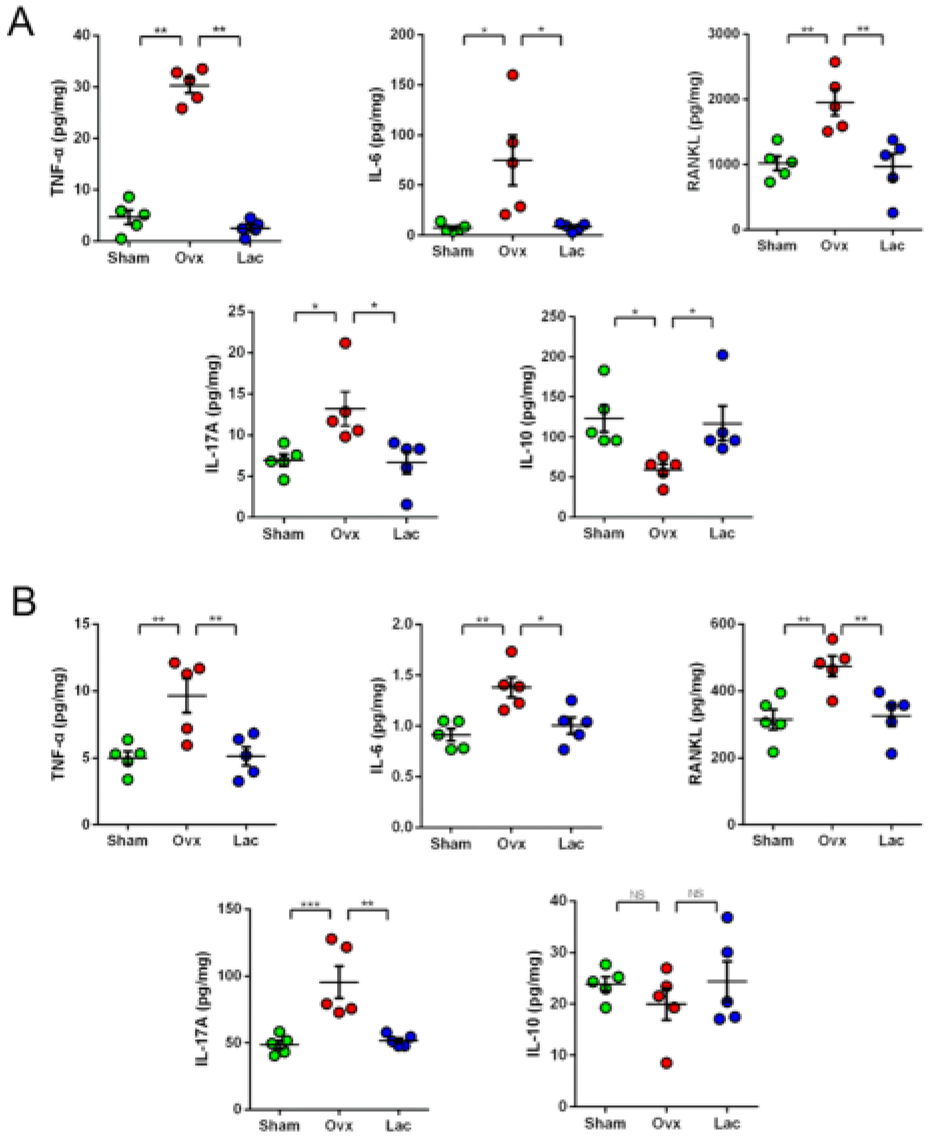

Supplementary Figure 5. **Lactulose inhibited pro-osteoclastogenic cytokines production in the bone marrow and peripheral blood.** (A-E) Levels of the inflammatory cytokines TNF-α, IL-6, RANKL, and IL-17 and anti-inflammatory cytokine IL-10 in the peripheral blood of Sham, OVX and OVX mice treated with lactulose. (F-K) Levels of the inflammatory cytokines TNF-α, IL-6, RANKL, and IL-17 and anti-inflammatory cytokine IL-10 in the bone marrow of Sham, OVX and OVX mice treated with lactulose. n = 5 mice per group in all panels. Data are expressed as mean ± SEM. **P* < 0.05, ***P* < 0.01, and ****P* < 0.001 compared with the corresponding group.

SUPPLEMENTARY DATA

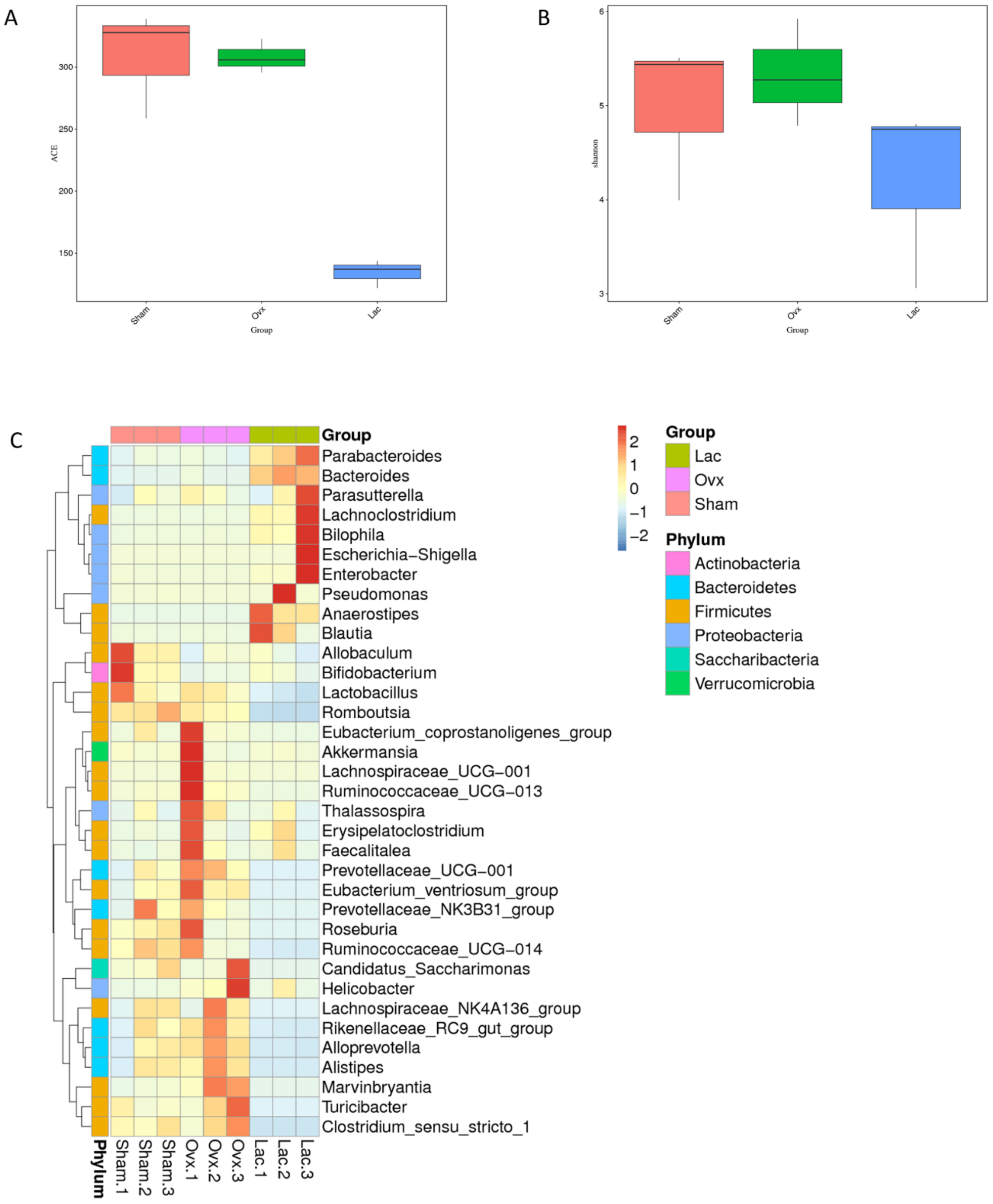

Supplementary Figure 6. 16Ss rDNA sequencing of feces and gas chromatography for SCFAs measurement. (A) ACE index (B) Shannon index (C) The phylogenetic relationships and the affiliated phylum for each genus. Data are expressed as mean ± SEM. **P* < 0.05, ***P* < 0.01, and ****P* < 0.001 compared with the corresponding group.

© 2019. Chen X et al. Published online at http://www.aginganddisease.org/EN/10.14336/AD.2019.0613
